# Supplementary material for: Rescue of imprinted genes by epigenome editing in human cellular models of Prader-Willi syndrome
Source: Nat Commun. 2025 Oct 28;16:9442. doi: 10.1038/s41467-025-64932-8 (PMC12569204; doi:10.1038/s41467-025-64932-8)
Supplement: Supplementary file 1 — Supplementary Information [file 41467_2025_64932_MOESM1_ESM.pdf]

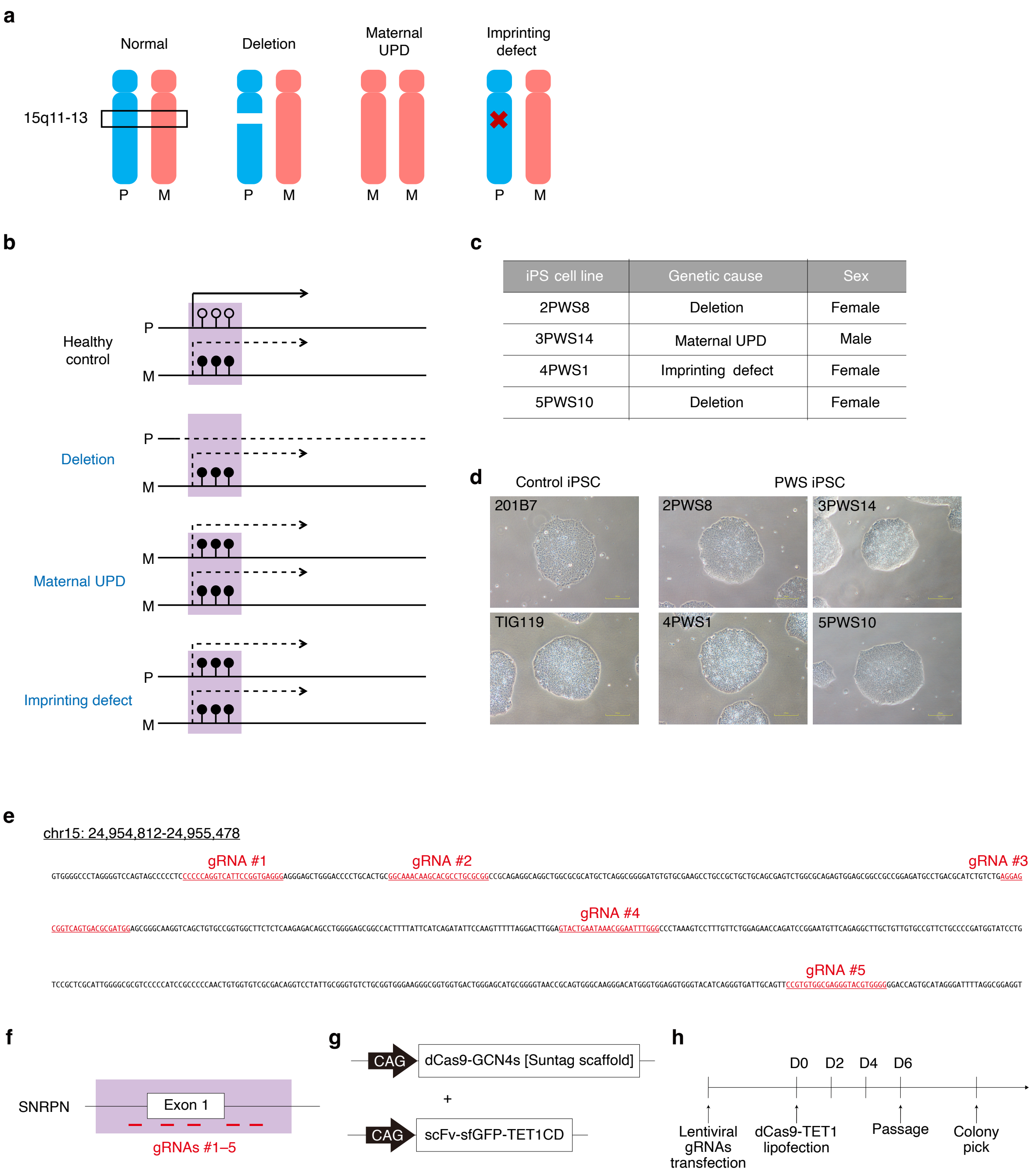

Supplementary Fig. 1: PWS iPSC lines and strategy of epigenome editing

a,**b**, Schematic illustrating PWS types. Deletion type has a deletion in paternal 15q11-13. Maternal uniparental disomy (UPD) type has two maternal chromosomes. Imprinting defect type has aberrant imprinting on the paternal chromosome. Blue and red indicate paternal and maternal chromosomes, respectively in (**a**). Black and white circles indicate methylated and unmethylated CpGs, respectively in (**b**). P, paternal chromosome; M, maternal chromosome.

c, iPSC lines derived from each type of PWS patient.

d, Representative bright-field images of PWS iPSC lines. Scale bar, 200 μm.

e,**f**, Sequences (**e**) and schematic (**f**) of genomic targets of gRNAs.

g, Transient overexpressing vectors for dCas9-Suntag-TET1 components. **h**, Schedule of epigenome editing of iPSCs.

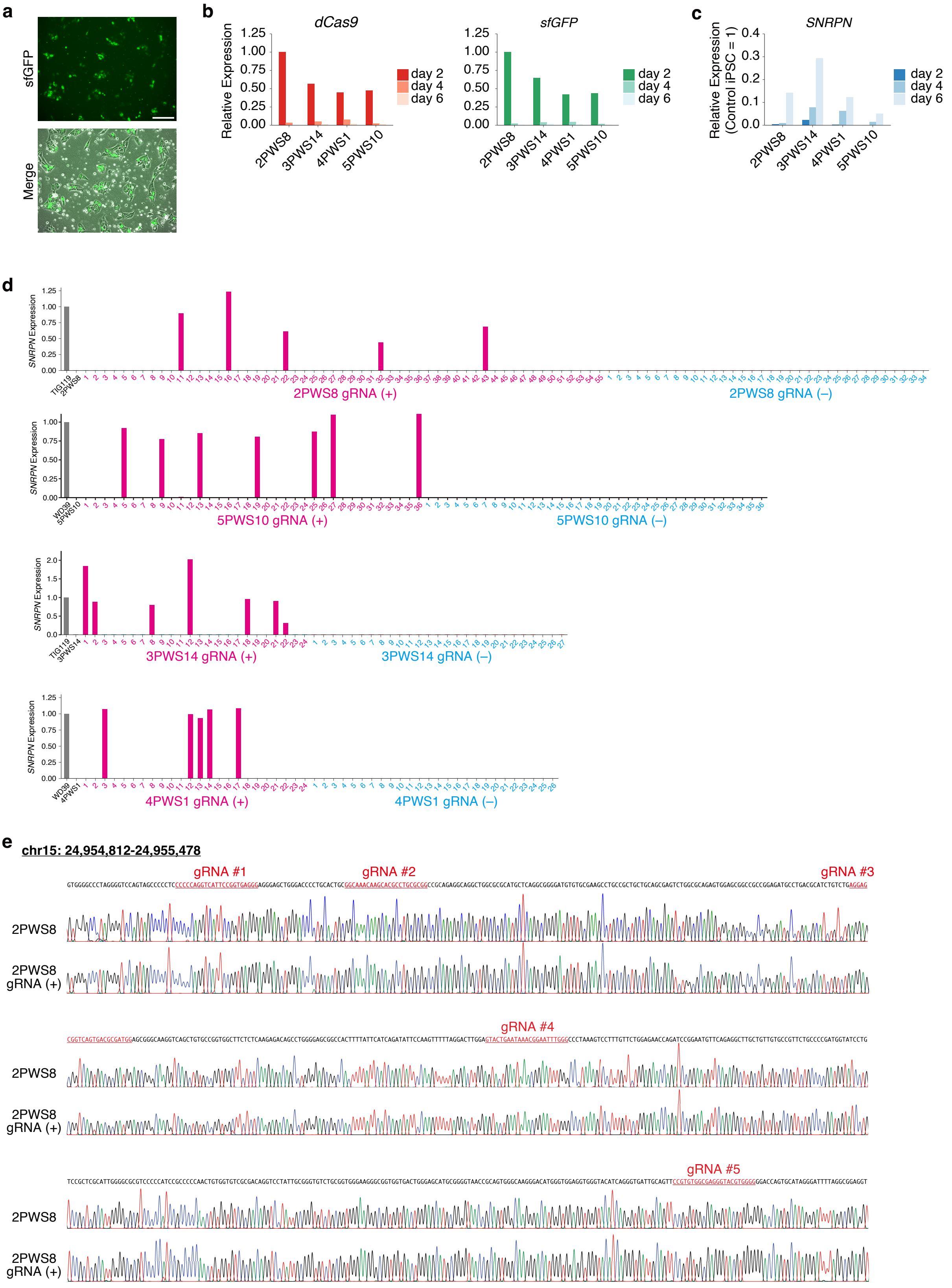

f

	TH01	D21S11	D5S818	D13S317	D7S820	D16S539	CSF1PO	AMEL	vWA	TPOX
2PWS8	6, 7	29	10, 11	11, 12	11	10, 13	10, 11	X	16, 18	8, 9
2PWS8 gRNA(+)	6, 7	29	10, 11	11, 12	11	10, 13	10, 11	X	16, 18	8, 9

Supplementary Fig. 2: Characterization of epigenome-edited iPSCs

a, Representative image of iPSCs transfected with dCas9-Suntag-TET1 components (dCas9-GCN4s and scFv-sfGFP-TET1CD). Scale bar, 200 μm.

b,**c**, RT-qPCR analysis of the expression of *dCas9*, *sfGFP* (**b**) and *SNRPN* (**c**) in iPSCs transfected with epigenome editing components.

d, RT-qPCR analysis of the expression of *SNRPN* in iPSC clones transfected with epigenome editing components.

e, Sanger sequence analysis of PWS-ICR in epigenome-edited iPSC clone and parental iPSC line.

f, Short tandem repeat analysis of epigenome-edited iPSC clone and parental iPSC line.

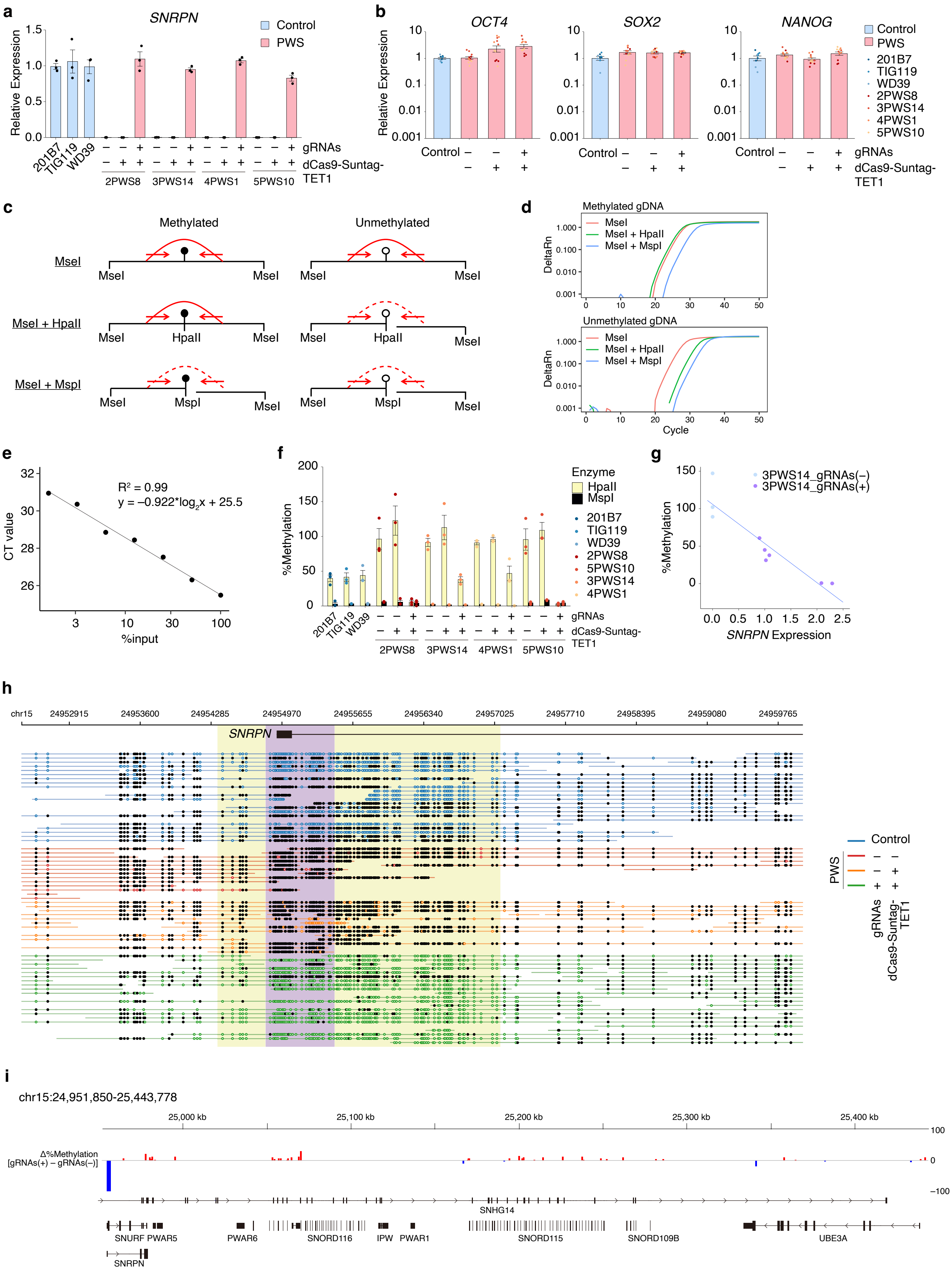

a
SNRPN
Control
PWS
Relative Expression
201B7
TIG119
WD39
gRNAs
dCas9-Suntag-TET1
2PWS8
3PWS14
4PWS1
5PWS10
b
OCT4
SOX2
NANOG
Control
PWS
201B7
TIG119
WD39
2PWS8
3PWS14
4PWS1
5PWS10
c
Methylated
Unmethylated
MseI
MseI + HpaII
MseI + MspI
HpaII
MspI
d
Methylated gDNA
Unmethylated gDNA
DeltaRn
Cycle
e
R² = 0.99
y = –0.922*log2x + 25.5
CT value
%input
f
Enzyme
HpaII
MspI
%Methylation
g
3PWS14_gRNAs(–)
3PWS14_gRNAs(+)
SNRPN Expression
h
chr15
24952915
24953600
24954285
24954970
24955655
24956340
24957025
24957710
24958395
24959080
24959765
SNRPN
Control
PWS
i
chr15:24,951,850-25,443,778
25,000 kb
25,100 kb
25,200 kb
25,300 kb
25,400 kb
Δ%Methylation [gRNAs(+) – gRNAs(–)]
SNHG14
SNURF PWAR5
PWAR6
SNORD116
IPW
PWAR1
SNORD115
SNORD109B
UBE3A
SNRPN

Supplementary Fig. 3: *SNRPN* expression and methylation change by epigenome editing

a, RT-qPCR analysis of *SNRPN* expression in control iPSCs, PWS iPSCs, and epigenome-edited PWS iPSCs (Control iPSCs [201B7, TIG119, WD39], n = 3 experiments, 1 iPSC line; PWS iPSCs [2PWS8, 3PWS14, 4PWS1, 5PWS10], gRNAs(–) dCas9-Suntag-TET1 (–), n = 3 experiments, 1 iPSC line; gRNAs(–) dCas9-Suntag-TET1 (+), n = 3 experiments, 3 clones from 1 iPSC line; gRNAs(+) dCas9-Suntag-TET1 (+), n = 3 experiments, 3 clones from 1 iPSC line). Control, 2PWS8, and 5PWS10 data are the same as Fig. 1b.

b, RT-qPCR analysis of the expression of *OCT4*, *SOX2*, and *NANOG* (Control iPSCs, n = 9 experiments, 3 iPSC line; gRNAs(–) dCas9-Suntag-TET1 (–), n = 12 experiments, 4 iPSC line; gRNAs(–) dCas9-Suntag-TET1 (+), n = 12 experiments, 12 clones from 4 iPSC line; gRNAs(+) dCas9-Suntag-TET1 (+), n = 12 experiments, 12 clones from 4 iPSC line).

c, Schematic illustrating genomic qPCR following methylation-sensitive restriction enzyme digestion. HpaII, a methylation-sensitive restriction enzyme, digests unmethylated CCGG sites, but not methylated CCGG sites. MspI is an isoschizomer of HpaII, but insensitive to methylation. HpaII digestion followed by PCR can amplify the PWS-ICR only when the CCGG site is methylated, while MspI-digested genomes cannot be amplified regardless of the methylation status. PCR amplification from undigested genomes is used as an internal control (100% methylation). MseI is used to fragment the genome for enhancing PCR efficiency, and there are no MseI recognition sites within PCR amplificated regions.

d, qPCR amplification curves of methylated (upper) and unmethylated (bottom) DNA. In methylated DNA, the MseI + HpaII-treated sample has a similar amplification to the MseI-treated sample, indicating the methylation of the CCGG site within the PWS-ICR. In unmethylated DNA, the MseI + HpaII-treated sample has a slower amplification than the MseI-treated sample, indicating the unmethylation.

e, A standard curve of genomic PCR amplification.

f, Methylation status of PWS-ICR in control iPSCs, PWS iPSCs, and epigenome-edited PWS iPSCs, measured by genomic qPCR analysis following methylation-sensitive enzyme digestion (Control iPSCs [201B7, TIG119, WD39], n = 3 experiments, 1 iPSC line; PWS iPSCs [2PWS8, 3PWS14, 4PWS1, 5PWS10], gRNAs(–) dCas9-Suntag-TET1 (–), n = 3 experiments, 1 iPSC line; gRNAs(–) dCas9-Suntag-TET1 (+), n = 3 experiments, 3 clones from 1 iPSC line; gRNAs(+) dCas9-Suntag-TET1 (+), n = 3 experiments, 3 clones from 1 iPSC line). Control, 2PWS8, and 5PWS10 data are the same as Fig. 1d.

g, Correlation between the methylation status of PWS-ICR and the SNRPN expression level in epigenome-edited 3PWS14 clones.

h, Per-read methylation status of PWS-ICR by long-read sequencing analysis. The purple highlighted region indicates the gRNA-targeted sites, and the yellow indicates differentially methylated regions.

i, Methylation difference between gRNAs(+) and gRNAs(–) iPSCs in the genomic region of *SNHG14*.

a

	20-mer match (On-target)	12-mer match	8-mer match
gRNA #1	1	3	838
gRNA #2	1	1	1,256
gRNA #3	1	0	132
gRNA #4	1	0	636
gRNA #5	1	0	726

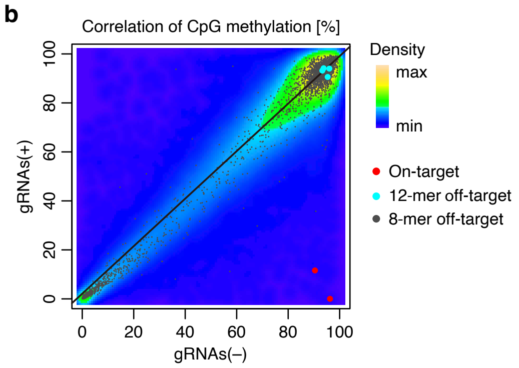

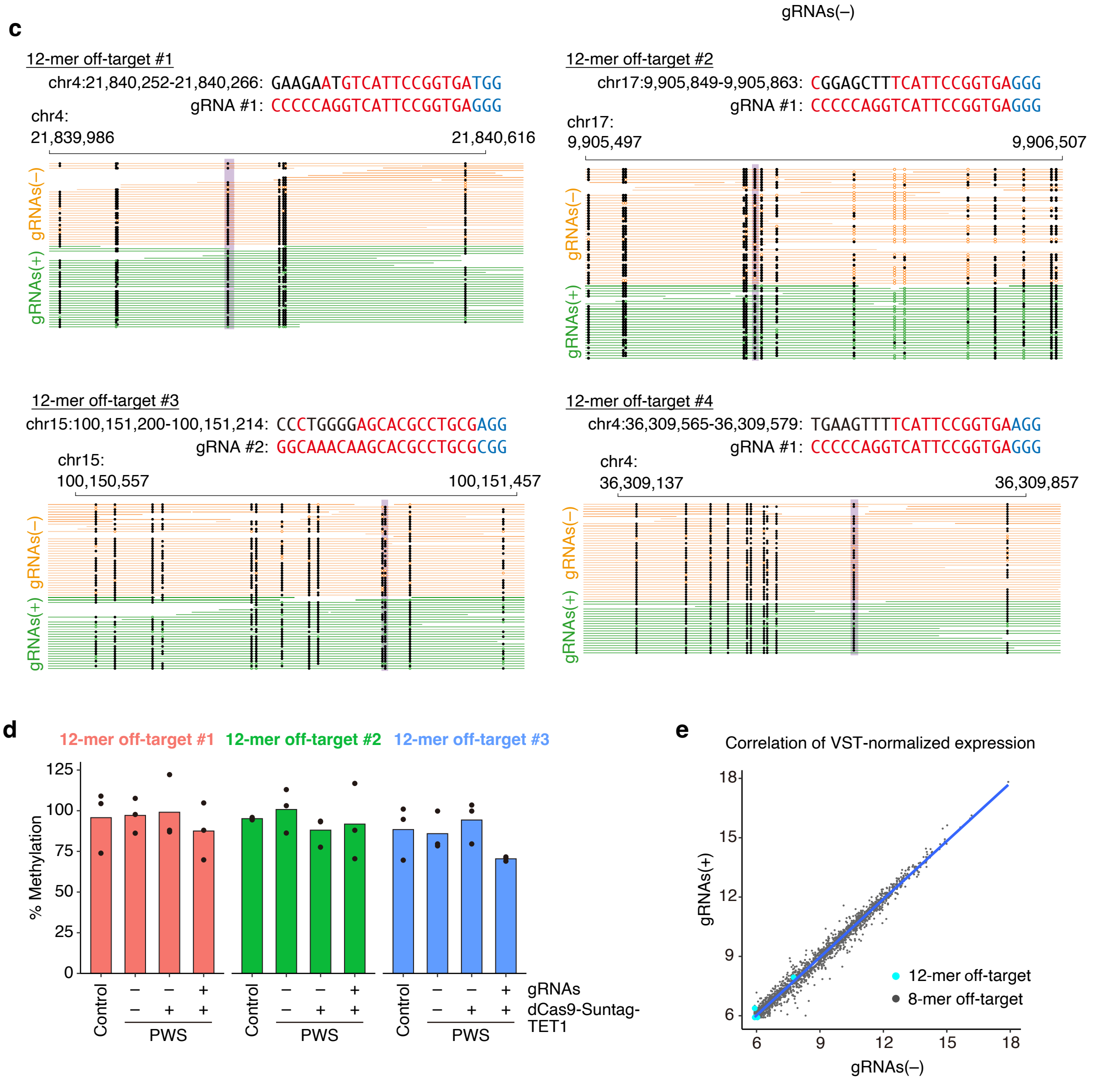

Supplementary Fig. 4: Off-target effects of epigenome editing

a, Potential off-target sites identified by CRISPRdirect based on the sequence homology with gRNA targets.

b, Correlation of CpG methylation between gRNAs(–) and gRNAs(+) iPSCs.

c, Per-read methylation status of potential off-target sites by long-read sequencing analysis. The purple highlighted region indicates the 12-mer off-target sites.

d, Methylation status of potential off-target sites in control iPSCs, PWS iPSCs, and epigenome-edited PWS iPSCs, measured by genomic qPCR analysis following methylation-sensitive enzyme digestion (Control, n = 3 experiments, 1 iPSC line; PWS iPSCs, gRNAs(–) dCas9-Suntag-TET1 (–), n = 3 experiments, 1 iPSC line; gRNAs(–) dCas9-Suntag-TET1 (+), n = 3 experiments, 3 clones from 1 iPSC line; gRNAs(+) dCas9-Suntag-TET1 (+), n = 3 experiments, 3 clones from 1 iPSC line).

e, Correlation of gene expression between gRNAs(–) and gRNAs(+) iPSCs.

a

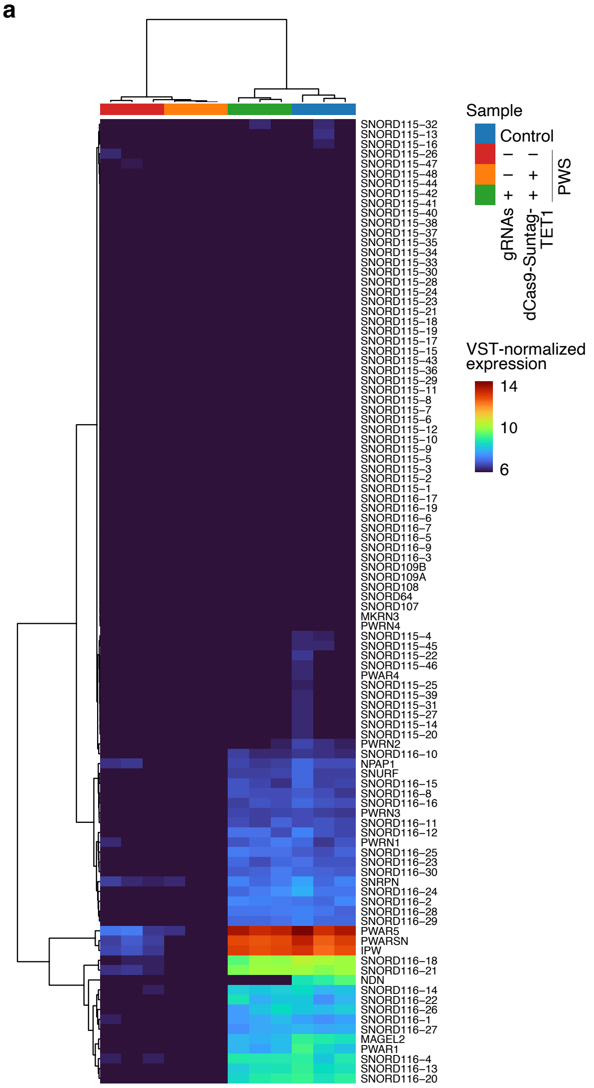

b

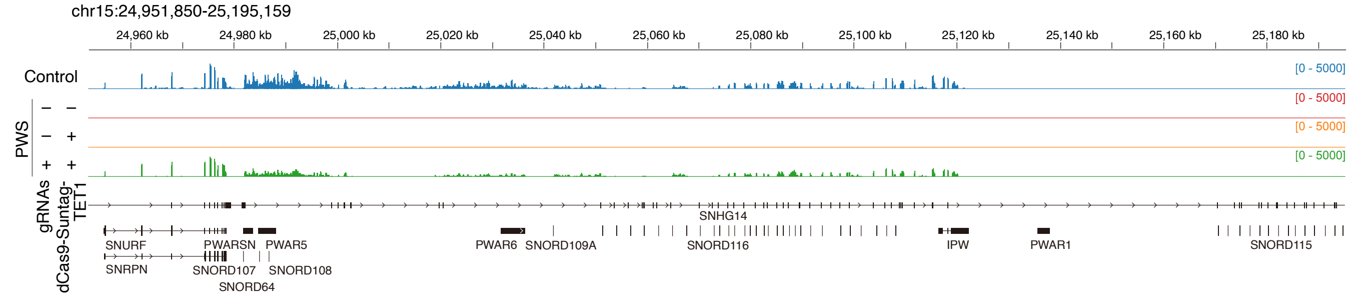

Supplementary Fig. 5: Transcriptomic analysis of epigenome-edited iPSCs
a, Heatmap summarizing the expression of PWS-associated imprinted genes.
b, RNA-seq read coverage mapped to the genomic region of *SNHG14*.

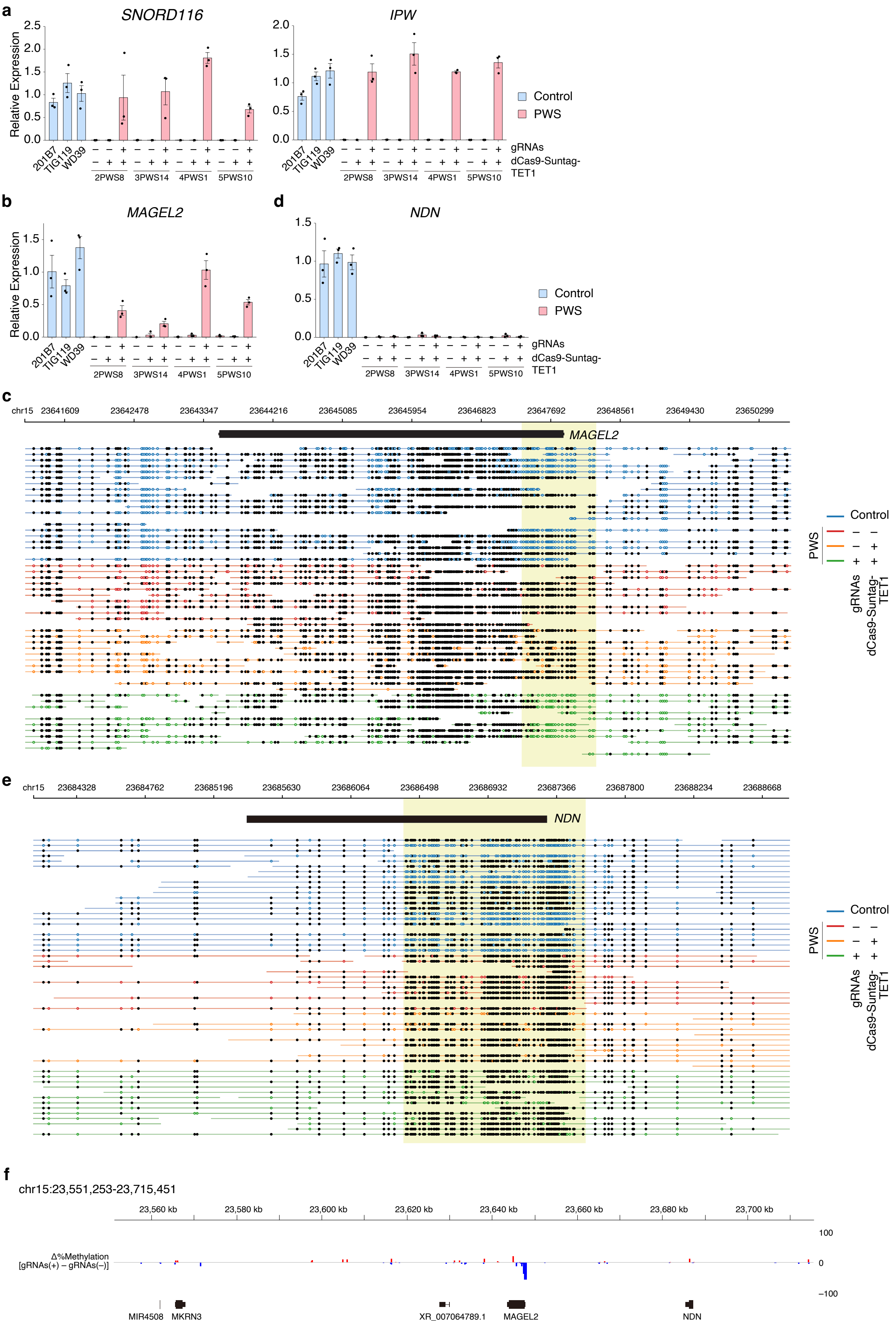
a
SNORD116
IPW
Relative Expression
2.5
2.0
1.5
1.0
0.5
0.0
201B7
TIG119
WD39
gRNAs
dCas9-Suntag-TET1
2PWS8
3PWS14
4PWS1
5PWS10
Control
PWS
b
MAGEL2
d
NDN
c
chr15
23641609
23642478
23643347
23644216
23645085
23645954
23646823
23647692
23648561
23649430
23650299
MAGEL2
e
23684328
23684762
23685196
23685630
23686064
23686498
23686932
23687366
23687800
23688234
23688668
NDN
f
chr15:23,551,253-23,715,451
23,560 kb
23,580 kb
23,600 kb
23,620 kb
23,640 kb
23,660 kb
23,680 kb
23,700 kb
Δ%Methylation
[gRNAs(+) – gRNAs(–)]
100
0
–100
MIR4508
MKRN3
XR_007064789.1
MAGEL2
NDN

Supplementary Fig. 6: Rescue of PWS-associated imprinted genes in epigenome-edited iPSCs

a,**b**,**d**, RT-qPCR analysis of the expression of *SNORD116* and *IPW* (**a**), *MAGEL2* (**b**), and *NDN* (**d**) in control iPSCs, PWS iPSCs, and epigenome-edited PWS iPSCs (Control iPSCs [201B7, TIG119, WD39], n = 3 experiments, 1 iPSC line; PWS iPSCs [2PWS8, 3PWS14, 4PWS1, 5PWS10], gRNAs(–) dCas9-Suntag-TET1 (–), n = 3 experiments, 1 iPSC line; gRNAs(–) dCas9-Suntag-TET1 (+), n = 3 experiments, 3 clones from 1 iPSC line; gRNAs(+) dCas9-Suntag-TET1 (+), n = 3 experiments, 3 clones from 1 iPSC line). Control, 2PWS8, and 5PWS10 data are the same as Fig. 1h,i,k.

c, Per-read methylation status of *MAGEL2* region by long-read sequencing analysis. The yellow highlighted region indicates differentially methylated regions in *MAGEL2* promoter.

e, Per-read methylation status of *NDN* region by long-read sequencing analysis. The yellow highlighted region indicates differentially methylated regions in *NDN* promoter.

f, Methylation difference between gRNAs(+) and gRNAs(–) iPSCs in the genomic region of *MKRN3–NDN*.

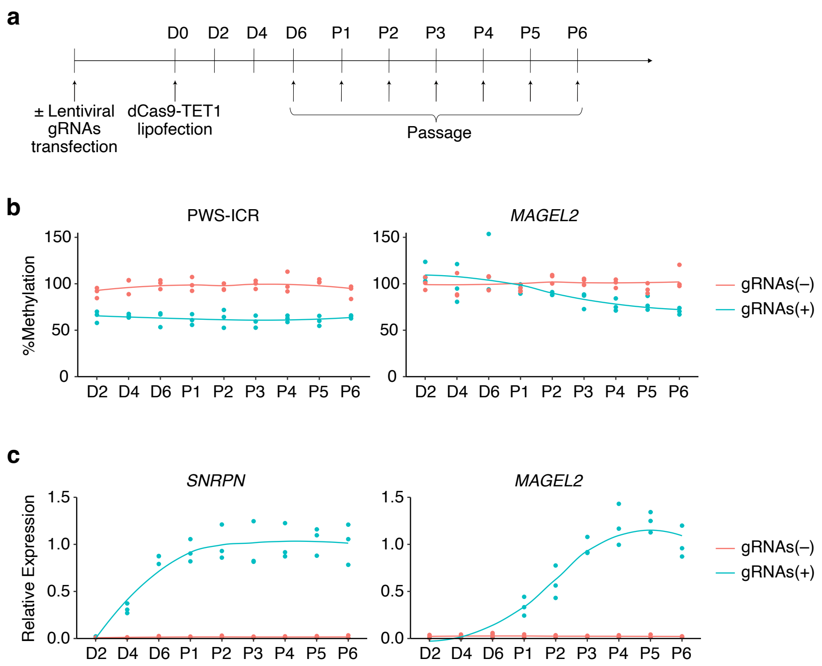

Supplementary Fig. 7: Methylation dynamics of PWS-ICR/*SNRPN* and *MAGEL2* during epigenome editing

a, Schedule of epigenome editing of iPSCs for methylation dynamics.

b, Methylation status of PWS-ICR and *MAGEL2* region in epigenome-edited PWS iPSCs measured by genomic qPCR analysis following methylation-sensitive enzyme digestion (gRNAs(–), n = 3 experiments, 1 iPSC line; gRNAs(+), n = 3 experiments, 1 iPSC line). Lines denote fitted curves by LOESS regression.

c, RT-qPCR analysis of the expression of *SNRPN* and *MAGEL2* (gRNAs(–), n = 3 experiments, 1 iPSC line; gRNAs(+), n = 3 experiments, 1 iPSC line). Lines denote fitted curves by LOESS regression.

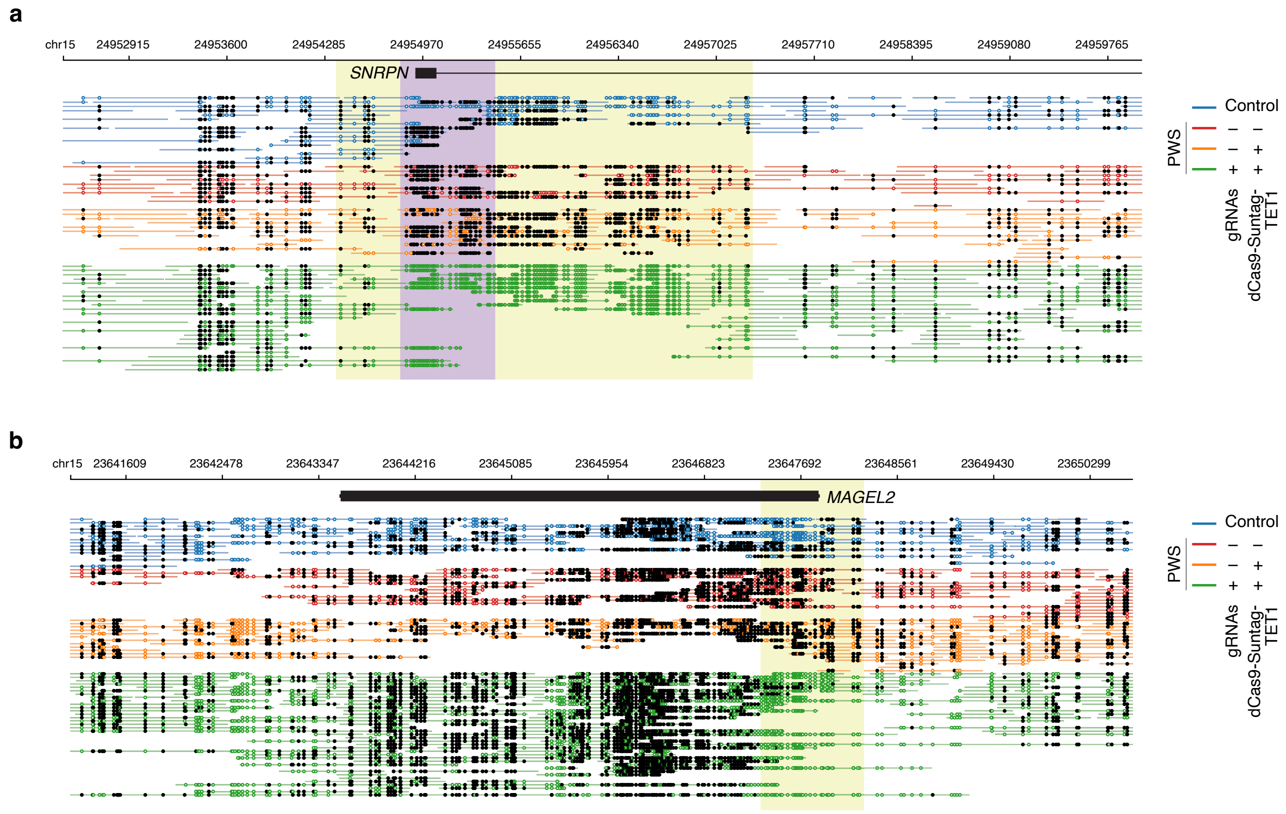

Supplementary Fig. 8: Nanopore long-read sequencing of imprinted genes in hypothalamic organoids

a,**b**, Per-read methylation status of PWS-ICR (**a**) and *MAGEL2* region (**b**) in organoids at day 100 by long-read sequencing analysis. The purple highlighted region indicates the gRNA-targeted sites, and the yellow indicates differentially methylated regions.

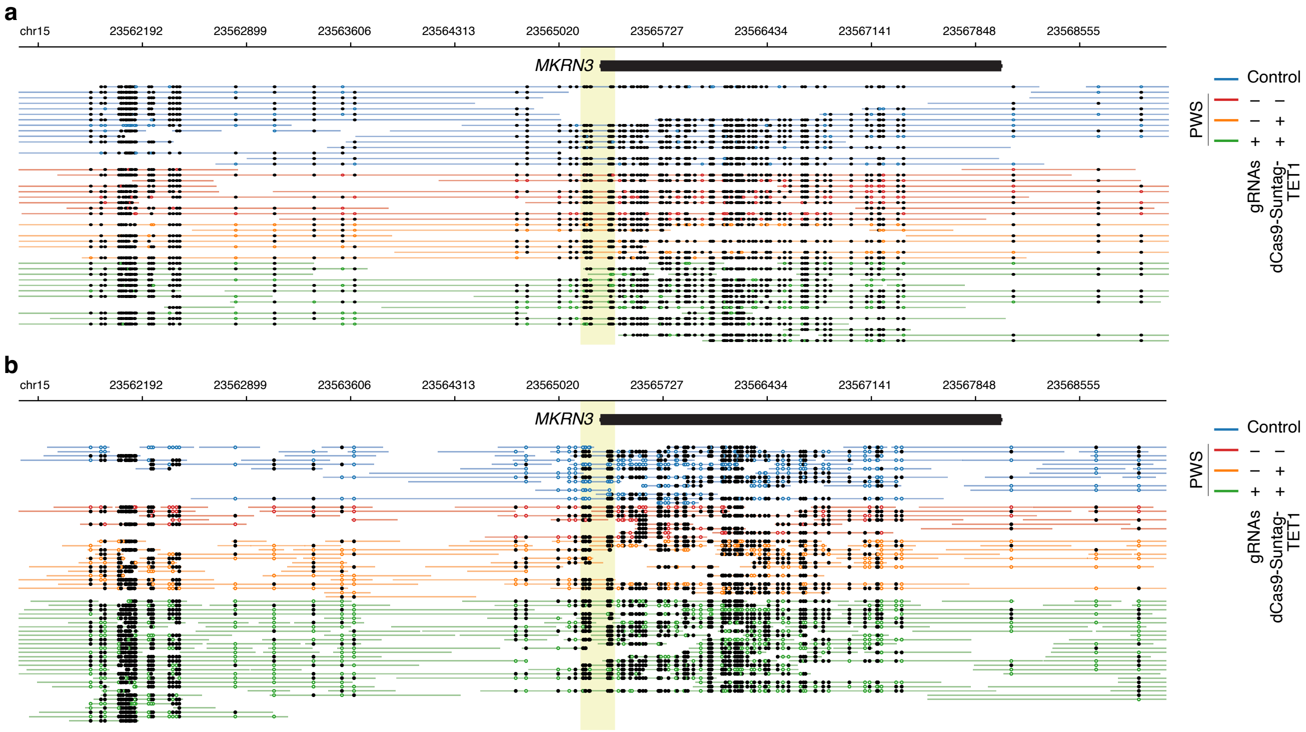

Supplementary Fig. 9: Nanopore long-read sequencing of *MKRN3* region

a,**b**, Per-read methylation status of *MKRN3* region in iPSCs (**a**) and organoids at day 100 (**b**) by long-read sequencing analysis. The yellow highlighted region indicates differentially methylated regions in *MKRN3* promoter.

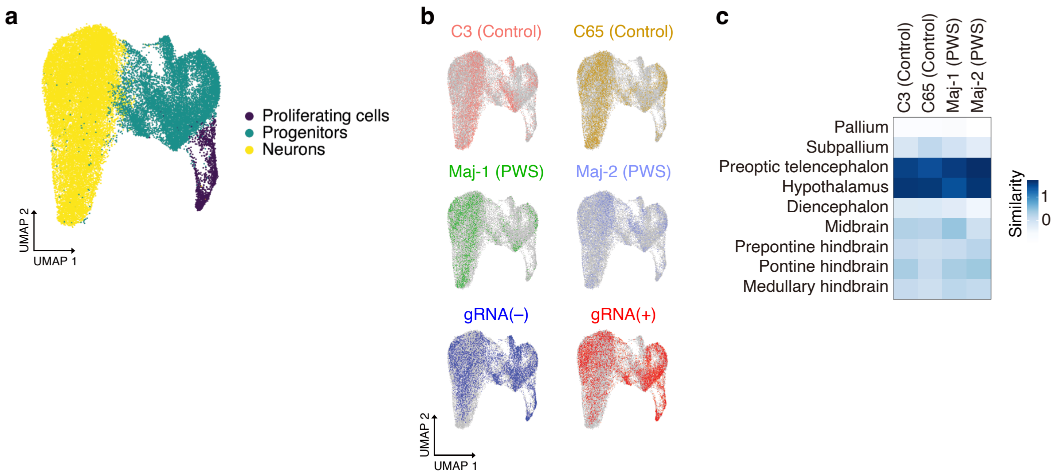

Supplementary Fig. 10: Single-cell RNA-seq analysis of iPSC-derived hypothalamic organoids
a, UMAP plot of scRNA-seq data integrated with a previous study (Huang et al., 2021) colored by cell type clusters.
b, UMAP plots colored by datasets. Cells in the two studies were distributed evenly, and there were no study-specific clusters.
c, VoxHunt spatial brain mapping of the samples of Huang et al., 2021 onto E15.5 mouse brains.

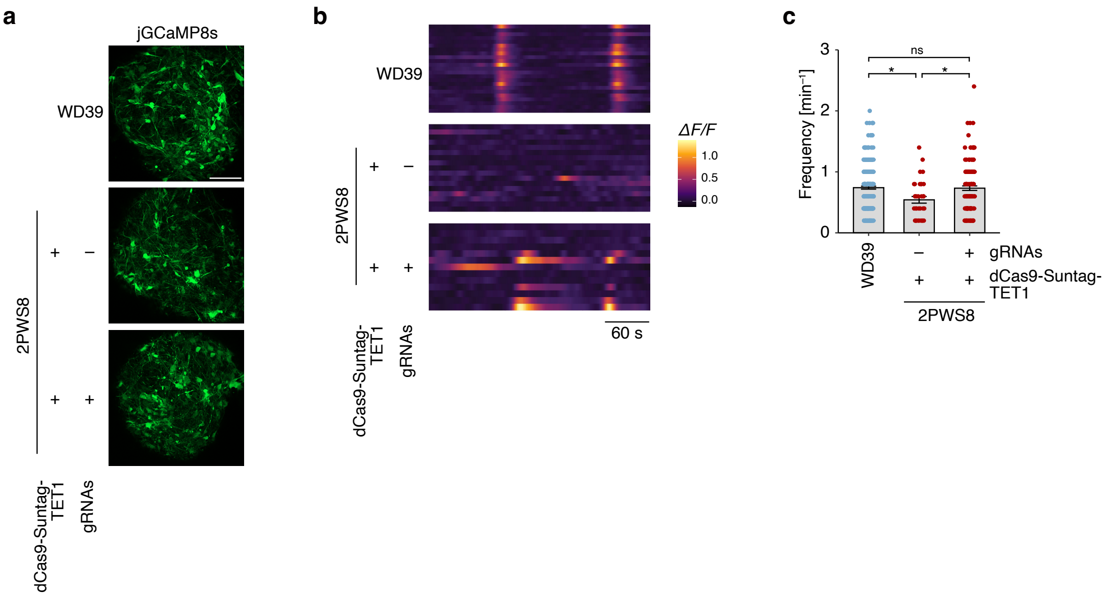

Supplementary Fig. 11: Calcium imaging of iPSC-derived hypothalamic organoids

a, jGCaMP8s-expressing organoids. Scale bar, 100 μm.

b, Representative heatmap of spontaneous calcium signal traces.

c, Frequency of spontaneous calcium activity (WD39, n = 267 cells from 5 organoids, 4 differentiation, 1 iPSC line; gRNAs(–) dCas9-Suntag-TET1 (+), n = 34 cells from 3 organoids, 3 differentiation, 1 clone from 1 iPSC line; gRNAs(+) dCas9-Suntag-TET1 (+), n = 133 cells from 3 organoids, 3 differentiation, 1 clone from 1 iPSC line; Kruskal–Wallis test with Dunn's correction, *P = 0.0137 [WD39 vs –/+], 0.0429 [–/+ vs +/+]; ns, not significant).

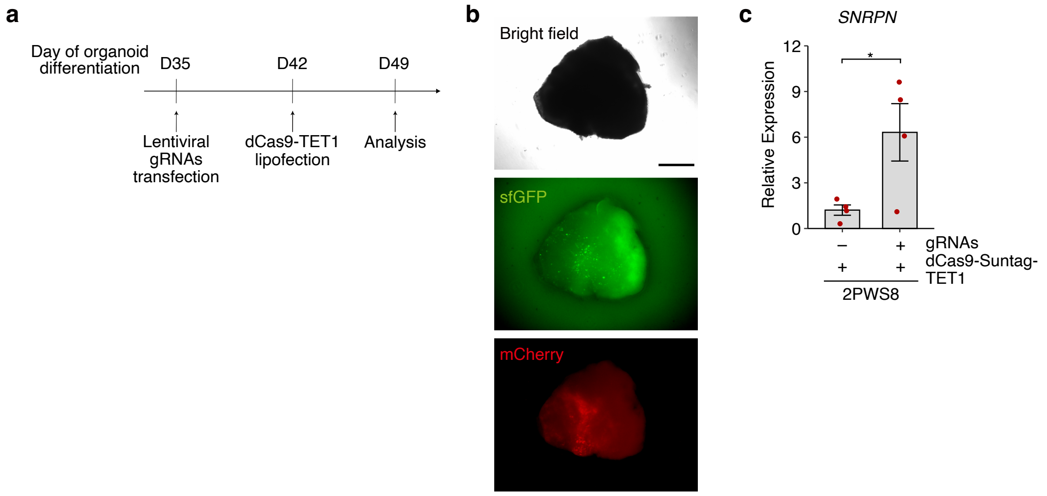

Supplementary Fig. 12: Epigenome editing in organoids

a, Schedule of epigenome editing of organoids.

b, Representative images of organoids transfected with mCherry-gRNAs and dCas9-Suntag-TET1 components (dCas9-GCN4s and scFv-sfGFP-TET1CD). Scale bar, 500 μm.

c, RT-qPCR analysis of *SNRPN* expression in organoids at day 49 (gRNAs(–) dCas9-Suntag-TET1 (+), n = 3 differentiation, 1 iPSC line; gRNAs(+) dCas9-Suntag-TET1 (+), n = 3 differentiations, 1 iPSC; unpaired t-test, *P = 0.0495).

Supplementary Table 1. Clones of epigenome-edited iPSCs used in this study

Figure	iPSC line	Clone of epigenome-edited iPSCs
1b, d, h, i, k	2PWS8	(gRNA–) 1, 2, 3; (gRNA+) 11, 16, 43
	5PWS10	(gRNA–) 1, 2, 3; (gRNA+) 5, 9, 13
1c	2PWS8	(gRNA–) 1, 2, 3; (gRNA+) 11, 16, 22
	5PWS10	(gRNA–) 1, 2, 3; (gRNA+) 5, 9, 13
1e	2PWS8	(gRNA–) 1; (gRNA+) 11
	5PWS10	(gRNA–) 1; (gRNA+) 5
1f, j, l	2PWS8	(gRNA–) 1; (gRNA+) 11
2e, f	2PWS8	(gRNA–) 1, 2, 3; (gRNA+) 11, 16, 43
2g	2PWS8	(gRNA–) 1; (gRNA+) 43
3b, c, d	2PWS8	(gRNA–) 1, 2, 3; (gRNA+) 11, 16, 43
3e	2PWS8	(gRNA–) 1; (gRNA+) 43
3f	2PWS8	(gRNA–) 1; (gRNA+) 11, 16, 43
4b	2PWS8	(gRNA–) 1; (gRNA+) 11
S3a, b, f	2PWS8	(gRNA–) 1, 2, 3; (gRNA+) 11, 16, 43
	3PWS14	(gRNA–) 1, 2, 3; (gRNA+) 2, 18, 21
	4PWS1	(gRNA–) 1, 2, 3; (gRNA+) 3, 12, 17
	5PWS10	(gRNA–) 1, 2, 3; (gRNA+) 5, 9, 13
S3g	3PWS14	(gRNA–) 1, 2, 3; (gRNA+) 1, 2, 8, 12, 18, 21
S3h	2PWS8	(gRNA–) 1; (gRNA+) 11
S3i	2PWS8	(gRNA–) 2; (gRNA+) 43
S4b, c	2PWS8	(gRNA–) 2; (gRNA+) 43
S4d	2PWS8	(gRNA–) 1, 2, 3; (gRNA+) 11, 16, 43
S4e	2PWS8	(gRNA–) 17, 21, 25; (gRNA+) 11, 16, 43
S5a, b	2PWS8	(gRNA–) 17, 21, 25; (gRNA+) 11, 16, 43
S6a, b, d	2PWS8	(gRNA–) 1, 2, 3; (gRNA+) 11, 16, 43
	3PWS14	(gRNA–) 1, 2, 3; (gRNA+) 2, 18, 21
	4PWS1	(gRNA–) 1, 2, 3; (gRNA+) 3, 12, 17
	5PWS10	(gRNA–) 1, 2, 3; (gRNA+) 5, 9, 13
S6c, e	2PWS8	(gRNA–) 1; (gRNA+) 11
S6f	2PWS8	(gRNA–) 2; (gRNA+) 43
S8a, b	2PWS8	(gRNA–) 1; (gRNA+) 43
S9a, b	2PWS8	(gRNA–) 1; (gRNA+) 43
S11a, b	2PWS8	(gRNA–) 1; (gRNA+) 16

Supplementary Table 2. List of RT-qPCR primers

Gene	Forward Primer	Reverse Primer
ACTB	TGAAGTGTGACGTGGACATC	GGAGGAGCAATGATCTTGAT
SNRPN	GGAAGTCCAAGTCAAACGC	CCTCGCTACTCCAATATGGC
SNORD116	TGGATCGATGATGAGTCC	TGGACCTCAGTTCCGATGAGA
IPW	TGCCTAGACCACCCACTAAAGG	AGTCTCCATGCGGAAGGAAGA
MAGEL2	CTGAGCCCGCAGAGTATGAG	GCGAGCGCTTCAAGGTAATG
NDN	TGTGTGTTGGGGTAGACTGC	AGTGTACTCCACGAGGGTGT
SNORD115	GTGTTGATGATGAGAACCTTATATTATCC	GGGCCTCAGCGTAATCCTATTG
UBE3A-ATS	GGAGAAACTGACACCACTCAA	CTCTTCCTCAGTCATCCTTATCAGA
UBE3A	GAGACTCAAAGTTAGACGTGACC	CCTTGTTCTCCTTCAAATTCCAC
MKRN3	GGGGTAACTGCCCATTTGGA	GTTGATGCCAGTATGCGCTG
dCas9	ACCATCGACCGGAAGAGGTA	CGATCCGTGTCTCGTACAGG
sfGFP	GTTCCGTGGCCAACACTTGTCACT	TACATAACCTTCGGGCATGGCACT
OCT4	GACAGGGGGAGGGGAGGAGCTAGG	CTTCCCTCCAACCAGTTGCCCCAAAC
SOX2	GGGAAATGGGAGGGGTGCAAAAGAGG	TTGCGTGAGTGTGGATGGGATTGGTG
NANOG	TGAACCTCAGCTACAAACAG	TGGTGGTAGGAAGAGTAAAG

Supplementary Table 3. List of methylation qPCR primers

Gene	Forward Primer	Reverse Primer
PWS-ICR (HpaII)	CATCTGTCTGAGGAGCGGTC	TCCAGAACAAAGGACTTTAGGG
MAGEL2 (HpaII)	GGCCAATGAAGCCTGCAAGTC	TATAGCCGCCCTACGGTTC
MKRN3 (HinP1I)	CTGCTAATGCCTTGCTGGTG	ATTCGTTCGGTTGACAAGGC
Off target #1 (HpyCH4IV)	GGGCACAACAGAAGAGAAACAGC	ACAACTGTGCTGAAACTCCACC
Off target #2 (HpaII)	AATGAAAGCTCCGTGAAGGATGG	GCCATGTGCCAGTTTCTAAGTGTG
Off target #3 (HpyCH4IV)	TGTTGTCTCCTACTGGCTGTTTC	AGGAGGTACAGGCATCCAGTG
