# Supplementary material for: Rescue of imprinted genes by epigenome editing in human cellular models of Prader-Willi syndrome
Source: Nat Commun. 2025 Oct 28;16:9442. doi: 10.1038/s41467-025-64932-8 (PMC12569204; doi:10.1038/s41467-025-64932-8)
Supplement: Supplementary file 3 — Reporting Summary [file 41467_2025_64932_MOESM3_ESM.pdf]

nature portfolio

Corresponding author(s): Hideyuki Okano and Hironobu Okuno

Last updated by author(s): Sep 18, 2025

Reporting Summary

Nature Portfolio wishes to improve the reproducibility of the work that we publish. This form provides structure for consistency and transparency in reporting. For further information on Nature Portfolio policies, see our Editorial Policies and the Editorial Policy Checklist.

Statistics

For all statistical analyses, confirm that the following items are present in the figure legend, table legend, main text, or Methods section.

n/a	Confirmed	
☐	☒	The exact sample size (*n*) for each experimental group/condition, given as a discrete number and unit of measurement
☐	☒	A statement on whether measurements were taken from distinct samples or whether the same sample was measured repeatedly
☐	☒	The statistical test(s) used AND whether they are one- or two-sided<br>*Only common tests should be described solely by name; describe more complex techniques in the Methods section.*
☒	☐	A description of all covariates tested
☐	☒	A description of any assumptions or corrections, such as tests of normality and adjustment for multiple comparisons
☐	☒	A full description of the statistical parameters including central tendency (e.g. means) or other basic estimates (e.g. regression coefficient) AND variation (e.g. standard deviation) or associated estimates of uncertainty (e.g. confidence intervals)
☐	☒	For null hypothesis testing, the test statistic (e.g. *F*, *t*, *r*) with confidence intervals, effect sizes, degrees of freedom and *P* value noted<br>*Give P values as exact values whenever suitable.*
☒	☐	For Bayesian analysis, information on the choice of priors and Markov chain Monte Carlo settings
☒	☐	For hierarchical and complex designs, identification of the appropriate level for tests and full reporting of outcomes
☒	☐	Estimates of effect sizes (e.g. Cohen's *d*, Pearson's *r*), indicating how they were calculated

Our web collection on statistics for biologists contains articles on many of the points above.

Software and code

Policy information about availability of computer code

Data collection	ZEN (Carl Zeiss) software was used for acquiring the microscopy data. ViiA7 Software (Thermo Fisher Scientific) was used to collect qPCR data. R9.4.1 and R10.4.1 flow cells were used for nanopore long-read sequencing on a GridION instrument (ONT).
Data analysis	The GraphPad Prism (V10.0.0) was used for statistical analyses. Fiji (v2.14.0) was used for image quantification and processing. Guppy and Megalodon were used for CpG methylation call in nanopore long-read sequencing. CellRanger (v7.0.1, 10x Genomics), R (v4.2.2), and Seurat (v4.3.0, R package) were used for single-cell RNA-seq analysis.

For manuscripts utilizing custom algorithms or software that are central to the research but not yet described in published literature, software must be made available to editors and reviewers. We strongly encourage code deposition in a community repository (e.g. GitHub). See the Nature Portfolio guidelines for submitting code & software for further information.

Data

Policy information about availability of data

All manuscripts must include a data availability statement. This statement should provide the following information, where applicable:

- Accession codes, unique identifiers, or web links for publicly available datasets
- A description of any restrictions on data availability
- For clinical datasets or third party data, please ensure that the statement adheres to our policy

Single cell RNA-seq data is available in GEO: accession number GSE262700.

Research involving human participants, their data, or biological material

Policy information about studies with human participants or human data. See also policy information about sex, gender (identity/presentation), and sexual orientation and race, ethnicity and racism.

Reporting on sex and gender	N/A
Reporting on race, ethnicity, or other socially relevant groupings	N/A
Population characteristics	N/A
Recruitment	N/A
Ethics oversight	N/A

Note that full information on the approval of the study protocol must also be provided in the manuscript.

Field-specific reporting

Please select the one below that is the best fit for your research. If you are not sure, read the appropriate sections before making your selection.

[x] Life sciences [ ] Behavioural & social sciences [ ] Ecological, evolutionary & environmental sciences

For a reference copy of the document with all sections, see nature.com/documents/nr-reporting-summary-flat.pdf

Life sciences study design

All studies must disclose on these points even when the disclosure is negative.

Sample size	We estimated sample sizes empirically based on previous studies (Okuno et al., 2017; Ideno et al., 2022).
Data exclusions	Cells with no activity events were not included in calcium imaging analysis.
Replication	qPCR and IHC data was acquired in at least 3 independent experiments. The data of bisulfite sequencing, nanopore long-read sequencing, and single-cell RNA-seq were obtained in one experiment.
Randomization	Samples were randomly picked from cell culture plates for each assay.
Blinding	Blinding was used for calcium imaging analysis. Blinding was not used or was not relevant for other experiments due to constraints in sample processing.

Reporting for specific materials, systems and methods

We require information from authors about some types of materials, experimental systems and methods used in many studies. Here, indicate whether each material, system or method listed is relevant to your study. If you are not sure if a list item applies to your research, read the appropriate section before selecting a response.

Materials & experimental systems

n/a	Involved in the study	
[ ]	[x]	Antibodies
[ ]	[x]	Eukaryotic cell lines
[x]	[ ]	Palaeontology and archaeology
[x]	[ ]	Animals and other organisms
[x]	[ ]	Clinical data
[x]	[ ]	Dual use research of concern
[x]	[ ]	Plants

Methods

n/a	Involved in the study	
[x]	[ ]	ChIP-seq
[x]	[ ]	Flow cytometry
[x]	[ ]	MRI-based neuroimaging

Antibodies

Antibodies used	Anti-RAX (guinea pig, TaKaRa Bio, M229, 1:500)<br>Anti-NKX2-1 (mouse, Thermo Fisher Scientific, MA5-13961, 1:50)<br>Anti-OTP (rabbit, GeneTex, GTX119601, 1:500)
Validation	Anti-RAX: This antibody has been used by previous studies, including Ideno et al., iScience, 2022.

Anti-NKX2-1: This antibody has been used by previous studies, including Imaizumi et al., Stem Cell Reports, 2015.
Anti-OTP: This antibody has been used by previous studies, including Rajamani et al., Cell Stem Cell, 2018.

Eukaryotic cell lines

Policy information about cell lines and Sex and Gender in Research

Cell line source(s)	Three human control iPSC lines (201B7, TIG119, and WD39) and one PWS line (2PWS8) were established in previous studies. Three PWS iPSC lines (3PWS14, 4PWS1, and 5PWS10) were newly established in this study from patients' blood cells with approval by the Keio University School of Medicine Ethics Committee.
Authentication	Karyotyping was performed with newly established cell lines.
Mycoplasma contamination	Mycroplasma contamination were routinely tested.
Commonly misidentified lines (See ICLAC register)	N/A

Plants

Seed stocks	*Report on the source of all seed stocks or other plant material used. If applicable, state the seed stock centre and catalogue number. If plant specimens were collected from the field, describe the collection location, date and sampling procedures.*
Novel plant genotypes	*Describe the methods by which all novel plant genotypes were produced. This includes those generated by transgenic approaches, gene editing, chemical/radiation-based mutagenesis and hybridization. For transgenic lines, describe the transformation method, the number of independent lines analyzed and the generation upon which experiments were performed. For gene-edited lines, describe the editor used, the endogenous sequence targeted for editing, the targeting guide RNA sequence (if applicable) and how the editor was applied.*
Authentication	*Describe any authentication procedures for each seed stock used or novel genotype generated. Describe any experiments used to assess the effect of a mutation and, where applicable, how potential secondary effects (e.g. second site T-DNA insertions, mosiacism, off-target gene editing) were examined.*
